# Dopamine and Dopamine-Related Ligands Can Bind Not Only to Dopamine Receptors

**DOI:** 10.3390/life12050606

**Published:** 2022-04-19

**Authors:** Jaromir Myslivecek

**Affiliations:** Institute of Physiology, 1st Faculty of Medicine, Charles University, Albertov 5, 128 00 Prague, Czech Republic; jmys@lf1.cuni.cz; Tel.: +420-224-968-485

**Keywords:** dopamine receptors, subtype selectivity, alpha-adrenoceptors, 5-HT receptors, antipsychotic drugs

## Abstract

The dopaminergic system is one of the most important neurotransmitter systems in the central nervous system (CNS). It acts mainly by activation of the D_1_-like receptor family at the target cell. Additionally, fine-tuning of the signal is achieved via pre-synaptic modulation by the D_2_-like receptor family. Some dopamine drugs (both agonists and antagonists) bind in addition to DRs also to α_2_-ARs and 5-HT receptors. Unfortunately, these compounds are often considered subtype(s) specific. Thus, it is important to consider the presence of these receptor subtypes in specific CNS areas as the function virtually elicited by one receptor type could be an effect of other—or the co-effect of multiple receptors. However, there are enough molecules with adequate specificity. In this review, we want to give an overview of the most common off-targets for established dopamine receptor ligands. To give an overall picture, we included a discussion on subtype selectivity. Molecules used as antipsychotic drugs are reviewed too. Therefore, we will summarize reported affinities and give an outline of molecules sufficiently specific for one or more subtypes (i.e., for subfamily), the presence of DR, α_2_-ARs, and 5-HT receptors in CNS areas, which could help avoid ambiguous results.

## 1. Introduction

The dopaminergic system is one of the most important neurotransmitter systems in the CNS. Dopamine receptors (DRs, see Abbreviations for abbreviation list) belong to G protein-coupled receptor (GPCR) family. According to their structural similarities, DRs are divided into two groups (for a review, see [1]): D_1_-like (D_1_ and D_5_ subtypes) and D_2_-like (D_2_, D_3_, and D_4_ subtypes). The families of DRs differ in the coupling to G proteins and subsequent steps of intracellular signalization. While D_1_-like DRs activate adenylyl cyclase via G_s_ protein, the D_2_-like family (mainly pre-synaptic D_2_ DRs) inhibits adenylyl cyclase via G_i_ protein activation. However, in detail, D_1_-like DRs activate not only adenylyl cyclase but also increase phosphoinositide metabolism [2]. Similarly, coupling with G_q_ protein allows D_2_ DRs to activate phospholipase C (see note about receptor variants below). D_1_-like receptors are characterized by non-simple interactions with various other mediators and receptor systems, which can be activity-dependent, comprise heterological oligomerization, dynamic compartmentalization of signaling components, and system integration for exquisite functional regulation (see [2] for detail). The adenylyl cyclase response is associated with the D_1_ subtype, while the phosphoinositide responses may be preferentially mediated through stimulation of the D_5_ receptor [2].

The genes for D_1_-like and D_2_-like families differ in the presence of introns in their coding sequence. While the D_1_-like family does not contain introns [3,4], the D_2_-like family does [5,6,7,8]. This fact allows the generation of receptor variants, “long” and “short” D_2_ receptor isoforms. These two isoforms exhibit largely similar pharmacological characteristics, but their differences in G protein coupling [9] suggest different functions [10].

### 1.1. D_1_-like Family

D_1_-like family is the main element of the dopamine post-synaptic action (despite its pre-synaptic localization). Its members, D_1_ and D_5_ DRs, are pharmacologically indistinguishable. However, the affinities of D_5_ DR to the agonists are up to 10 times higher than that of D_1_ ones [11]. This fact could be of importance when one transmitter is supposed to have two effects—one through the high-affinity sites and the second one through the low-affinity sites in tissue expressing both subtypes. This could explain the different functions of striatal D_1_ and D_5_ DRs in synaptic plasticity [12]. Another difference between these two subtypes that is interesting to mention is that the D_5_ dopamine receptor, unlike the D_1_ subtype, is constitutively (agonist-independently) active [13]. Moreover, D_1_ DRs couple preferentially to G protein heterotrimers that contain γ7 subunits [14]. D_1_ DRs can also couple to another G protein, G_olf_ (which also stimulates adenylyl cyclase) that is highly expressed in some brain areas, such as the caudate nucleus, nucleus accumbens, and olfactory tubercle. Some coupling of D_1_ DR with G_olf_ was even suggested to be preferential [15]. The generation of D_5_ DR knockout mouse uncovered possible involvement of this subtype in the pathology of hypertension, as the mutant mice were hypertensive [16].

### 1.2. D_2_-like Family

D_2_ DRs are as D_1_ DRs [17] localized both pre- and postsynaptically. D_2_ DR has a relatively low (nanomolar) affinity for dopamine, which supports its importance as a modulatory (pre-synaptic) receptor. D_2_ DR isoforms (long and short) are differently distributed and thus may possess distinct functions. The short isoform seems to serve as an autoreceptor, whereas the long isoform is primarily a post-synaptic receptor [18]. Using genetically targeted deletion of the D_2_ dopamine receptor gene in mice revealed that other members of the receptor family were not affected [19] and these mutants had reduced locomotion and less coordinated movement [19].

D_3_ subtype of DR appears to have similar distribution as the D_2_ dopamine receptor [1]. Similar to D_2_ DR, alternative splicing variants of D_3_ DR were observed. These variants were hypothesized to contribute to the availability of active D_3_ DRs in some psychiatric conditions [20]. This hypothesis suggests that inactive D_3_ DRs affect ligand binding to the active D_3_ DRs and thus influence their function. 

The D_4_ DR has high densities in the cerebral cortex, amygdala, hypothalamus, and pituitary [21]. In the striatum, the occurrence of the D_4_ DR is much lower than the D_1_ and D_2_ subtypes [22].

### 1.3. DR Ligand Targets

We have described above that signaling through DRs is far from to be simple. What is more, some DR ligands bind not only to DRs, but the spectrum of targets is much wider. Surprisingly, this is valid for dopamine itself. This natural neurotransmitter binds not only to DRs (D_1_-D_5_ pK_i_s [see Abbreviations for abbreviation list and the elucidation of differences between pK_i_ and pEC_50_ in the next paragraph] vary between 4.3–7.6 [7,8,23]), and dopamine transporter (DAT, pK_i_ = 5.3 [24]) but also to other transporters (norepinephrine transporter—NET (pK_i_ = 4.55 [25]), serotonin transporter—SERT, pK_i_ = 4.53 [25]), to other receptors (α_1_-ARs (pKi-5.6, [26]), α_2_-ARs (pK_i_ = 6.01, [26]), β_1_-, β_2_-ARs (pK_i_ = 5.0, pK_i_ = 4.3, respectively [27]) and to melatonin receptors MT_1A, 1B_, pK_i_ = 5.15, pK_i_ = 5.04, respectively). Looking at these numbers, it is possible to conclude that dopamine is bound with a similar affinity to D_1_ and D_2_ DRs (pK_i_ = 4.3–5.6, pK_i_ = 5.3–6.4, respectively) and DAT, NET, SERT, α_1_-, and α_2_-ARs, β_1_-, β_2_-ARs, and to melatonin receptors MT_1A, 1B_ (see the pK_i_s above). Other DRs have to dopamine a higher affinity (pK_i_ = 6.3–7.4, 7.6, 6.6, respectively, for D_3_, D_4_, and D_5_ DRs).

It is necessary to mention (please see the values in this review) that binding assessed parameters (i.e., pK_i_s) differ from the values determined using functional studies (i.e., dose-response determined constants, pEC_50_s [28]). This is because in studies based on dose-response determined parameters; the ligand usually discards the presence of other receptors on the studied effect by a combination of pharmacological means to attribute properly the receptor involved. Another possibility is that in dose-response studies, the formation of a ligand-receptor complex with activation of G protein and further with target second messenger producer activation is more complicated than the binding of ligand to the receptor in binding studies. The interesting correlation between pK_i_ and pEC_50_ has been demonstrated for neurokinin NK_1_ receptors [29]. Although this is a specific example for specific receptors and specific ligands, we can assume that a similar correlation can be found for DRs and their ligands too. As reported here, the pK_i_s and pEC_50_s differ for D_1_-like DR to SKF 38393. With some methodological reservation, one could construct the correlation between these values reported in [13,23,30,31,32,33,34] in humans and rats.

A similar multitarget binding can be found for DR agonists and antagonists. This review will focus on such interactions that can broaden the physiological effects elicited by dopamine ligands in the central nervous system. Besides, these interactions could present the potential problem with results interpretation: the ligand activating more neurotransmitter receptors that have similar affinity to them can distort the conclusions made. With this point of view, this review could help with careful interpretation of the results obtained. We will focus on orthosteric binding sites only, although there are also described allosteric binding sites on D_2_ DR [35]. The allosteric binding sites [36,37] and their interaction with other molecules exceed the topics of this review. The inclusion criteria were the ability to bind to other targets with pK_i_ ≥ 7.0, pK_IC50_ ≥ 7.0 if the pK_i_ for DRs is between 8 and 9. Interestingly, some papers report a surprisingly high concentration of drugs used as proof of specific dopamine subtype involvement even though the selectivity of such ligand is limited (e.g., SKF 38393 in concentration 100 μmol/L affects all dopamine receptor subtypes and also α_2C_-AR). If the ligand is sufficiently specific to dopamine receptors (i.e., the affinity differs at least two orders of magnitude), then it is not reviewed here.

The interested researcher should search available databases carefully for the ligands with well-documented selectivity to specific DR subtypes and not rely on the information from the manufacturer. The specific ligand should be at least two orders of magnitude more specific for the respective DR subtype than to the others. In other words: ΔpK_i_s(pK_i1,_ pK_i2_) ≥ 2. The examples of such ligands are shown in Table 1. On the other hand, the new research can bring new knowledge, and the supposed selectivity of the specific ligand could be doubted. Thus, it is necessary, before the choice of ligand, carefully check the present knowledge to avoid the use of non-specific ligands.

When using radioligand for receptor detection, one should be aware that a better option is to use an antagonist than an agonist because of stronger binding and lower possibility of dissociation of such ligand from the receptors.

## 2. DR Agonists

### 2.1. So-Called Selective Dopamine Receptor Agonists

The typical problem with dopamine ligand lies in the fact that manufacturers usually declare the ligand as selective, which could be, in some cases, far from reality. This could be misleading, and it could distort the conclusions made with such a “selective” drug. In the following paragraphs, we will describe the DR agonist in which the selectivity is limited. Other ligands that are selective according to present knowledge will not be mentioned. 

We can generalize that dopamine drugs (both agonists and antagonists) bind in addition to DRs also to α_2_-ARs and 5-HT receptors. Thus, it is important to consider the presence of these receptor subtypes in specific CNS areas as the function virtually elicited by one receptor type could be the effect of other—or the co-effect of multiple receptors. The presence of neurotransmitter receptors in the CNS is shown in Table 2. In addition to that, dopamine ligands often bind to H_1_ histamine receptors. These receptors are present in many CNS structures [39]: cerebral cortex, hippocampal dentate gyrus, amygdaloid complex, basal forebrain, nucleus accumbens, islands of Calleja, septal nuclei, thalamus, hypothalamus (medial preoptic area, dorsomedial, ventromedial, and most posterior nuclei, including the tuberomammillary complex), nuclei of origin of most cranial nerves, and in the dorsal horn of spinal cord.

As an example, we can use SKF 38393. One of the manufacturers claims that this is a prototypical D_1_-like DR selective partial agonist. The careful search for pK_i_ values (pEC_50_ values, respectively, see the discounts in Section 1.3), however, can indicate pK_i_ = 6.41–6.8 [13,23,30] in human, pK_i_ = 7.19 in rat [31], pEC_50_ = 5.0–8.96 in human for D_1_ DR [32,34], pK_i_ = 6.91–7.0 for D_5_ DR in human [23,33], and pK_i_ = 5.16 for D_2_ DR in rat [31]. These values indicate selectivity to D_1_-like DRs, but still show some effect on D_2_ DR. More importantly, SKF 38393 is also bound by α_2C_-AR with pKi = 7.08 [45], i.e., in the rank in which D_1_ and D_5_ DRs are activated.

This is important in tissues in which are DRs and ARs co-expressed (see Table 2). D_1_-like DRs are present [40] together with α_2C_-ARs [41] in the following brain areas: the cerebral cortex and amygdala. In general, α_2C_-ARs presence is described in the basal ganglia, and D_1_ DRs are abundantly present in the subthalamic nucleus and caudate-putamen. The D_2_ DRs (although they have a lower affinity to SKF 38393) are simultaneously present in α_2C_-ARs in the substantia nigra pars compacta and the ventral tegmental area. In those brain areas, one should be careful when interpreting the results obtained with SKF 38393 as both effects on DRs and α_2_-ARs can be present. Ignoring the fact that SKF 38393 activates D_1_-like DRs and blocks α_2C_-ARs could lead to misinterpretation of the results.

Another “selective” D_1_ DR ligand is the partial agonist A68930, although also designated as sub-family selective. This compound was reported to have a similar effect on rat D_1_ and D_5_ DRs (pEC_50_ = 6.82 and 6.6, respectively, [46]). The other data showed higher pEC_50_ at D_1_ DRs in the rat (pEC_50_ = 8.71, when pK_i_ = 8.8 [47]). This study also determined pK_i_ = 6.09, and pEC_50_ = 4.99 at D_2_ DRs in the rat. This drug also binds to 5-HT_1A_, 5-HT_2C_ serotonin receptors, and β_1_-ARs with pK_i_ = 5.59, 5.0, and 5.0, respectively [47]. Although the affinity of 5-HT_1A_, 5-HT_2C_ serotonin receptors, and β_1_-ARs is lower than D_1_-like DRs (when considering the data from [47]), the data from [46] are quite similar, and one should be cautious with the interpretation of the results obtained with this drug.

Quinpirole is very often declared by manufacturers as a selective dopamine D_2_ DR (or D_2_-like) agonist. As an example, quinpirole sensitization was used as a model of obsessive-compulsive disorder [48], targeting the D_2_ and D_3_ DRs. However, the pK_i_ values for D_2_, D_3_, D_4_, and D_1_ DRs, respectively (pK_i_ = 4.9–7.7 [49], pK_i_ = 7.3–7.7 [49], pK_i_ = 7.5 [50], pK_i_ = 4.06–7.2 [51,52], respectively) do not reveal the full selectivity. The spectrum of quinpirole action is much wider: 5-HT_2B_, 5-HT_2A_, and 5-HT_2C_ receptors reveal pK_i_ = 5.0–6.5 [50], and 5-HT_1A_ receptor reveal pK_i_ = 5.8 [53]. These values are apparently in the rank of DR action. Quinpirole also produces significant THC-like effects when metabolic degradation of anandamide is inhibited, supporting the hypothesis that these effects of quinpirole are mediated by cannabinoid CB1 receptors [54].

Sumanirole (PNU-95,666) is assumed as a highly selective D_2_ DR full agonist, the first of its kind to be discovered [55] with D_2_ DR pK_i_ = 8.1 [56]. 5-HT_1A_ receptor reveals pK_i_ = 7.14 [57] to sumanirole, which is too close to the pK_i_ for D_2_ DR and co-effect should exist. There is also agonist activity of sumanirole at human D_3_ DR transfected in HEK293T cells, revealing pK_i_ = 6.73 [58], suggesting slightly limited selectivity of sumanirole on D_2_ DR. It means that 50% of D_2_ DRs are occupied by approximately 8 nmol/L sumanirole and 50% of D_3_ DRs are occupied by approximately 189 nmol/L sumanirole. 20 nmol/L should completely block D_2_ DRs, but also 10% of D_3_ DRs. 

### 2.2. Drugs–Dopamine Receptor Agonists with Multiple Targets of Action 

Usually, the drugs used in the treatment have multiple targets of action, which can be an advantage as multiple targets are affected by one drug. In the following paragraphs, we will mention the drugs that: (1) also have DRs action, (2) are declared as a drug with multiple targets. This could help in the interpretation of the effects obtained with this drug that could be erroneously attributed to one target only.

An example of such a drug is fenoldopam, which causes arterial/arteriolar vasodilation decreasing blood pressure. Fenoldopam is used for the in-hospital, short-term (up to 48 h) management of severe hypertension, including malignant hypertension. It is declared as an agonist for D_1_ DRs with moderate affinity to α_2_-ARs and no significant affinity for D_2_ DRs, α_1_ and β-ARs, 5-HT_1_ and 5-HT_2_ receptors, or muscarinic receptors. 

However, fenoldopam is also bound with similar affinity to D_5_ DR (pK_i_ = 9.1 for D_1_ DR, pK_i_ = 9.2 for D_5_ DR, respectively) and D_2_ DR (pK_i_ = 8.5), and with lower affinity to D_4_ DR (pK_i_ = 6.8) [59]. Some data indicate pK_i_ to D_2_ DR is lower (4.89–5.89, [60]). Early evidence showed that fenoldopam had no effect on β-ARs, but had antagonistic activity on α_1_-ARs [61] (pA2 = 8.36 ± 0.21), although in some papers characterized as weak (pK_i_ = 5.41, [62], or modest pK_i_ = 6.82 [26]) and α_2_-ARs [63] (pK_i_ = 7.60–7.78, [62]). Fenoldopam thus represents the typical multiple targets drug. This is a disadvantage with respect to the specific effect of receptors when aiming to determine the subtype involved in the function but could be an advantage when targeting to specific therapeutic aim (e.g., acute severe hypertension treatment).

Another example of a drug with multitarget action is atypical antipsychotic aripiprazole. This drug acts as an atypical agonist on D_2_ DRs (pK_i_ = 9.7 [64]) with expressed selectivity over D_4_ DRs (pK_i_ = 7.3 [64]). However, on D_4_ DRs its action is antagonistic. The multitargeting of this ligand comprises partial agonism on 5-HT_1A_ and 5-HT_2A_ serotonin receptors with pK_i_ = 8.2 [65], and pK_i_ = 7.5–8.1 [65], respectively. On 5-HT_1D_ aripiprazole reveals full agonism with pK_i_ = 7.2 [65]. Other serotonin receptors affected by aripiprazole are 5-HT_7_ (partial agonism, pK_i_ = 7.8 [66]) and 5-HT_2C_ (partial agonism, pK_i_ = 7.6 [67]). H_1_ histamine receptors are antagonized by this ligand with pK_i_ = 7.5 [67].

A wide spectrum of action also reveals cabergoline which is an ergot-derived, long-acting D_2_ DR agonist and prolactin inhibitor. However, the D_2_ DR selectivity is rather declared than it corresponds to the reality. This drug binds, besides to DRs, to other receptor proteins [50]: D_2_ DRs and D_3_ DRs bind this drug with similar affinity as a partial agonist (pK_i_ = 9.0–9.2, and pK_i_ = 9.1 for D_2_ DR and D_3_ DR, respectively), similar affinity reveal 5-HT_2B_ receptors (pK_i_ = 8.9, full agonist) and very close affinity show 5-HT_2A_ and 5-HT_1D_ (pK_i_ = 8.2 and pK_i_ = 8.1, respectively for 5-HT_2A_ (full agonist) and 5-HT_1D_ receptors [partial agonist]). On the other D_2_-like DRs (D_4_ DR) it also behaves as a partial agonist, but the affinity is lower (pK_i_ = 7.3). Besides these effects cabergoline acts also as an antagonist on α_2A_-AR, α_2C_-AR, α_2B_-AR, and α_1A_-AR (with pK_i_ = 7.9, pK_i_ = 7.7, pK_i_ = 7.1, and pK_i_ = 7.1, respectively on α_2A_-AR, α_2C_-AR, α_2B_-AR, and α_1A_-AR) and as a full agonist on 5-HT_1A_ receptor (pK_i_ = 7.7) [50]. One should be cautious when thinking about the D_2_ DR or D_2_-like selectivity. Although about 1.5 order of magnitude difference (pK_i_ about 9.0 for D_2_ DRs), the affinity of D_1_-like receptors could still play a role in the action of cabergoline: on D_5_ DR it behaves like a full agonist with pK_i_ = 7.7, on the D_1_ DR it reveals a similar type of action (full agonism), but the pK_i_ = 6.7 is significantly lower [50]. The affinity (full agonism) of 5-HT_1B_ and 5-HT_2C_ is much lower than the affinity of other receptors (pK_i_ = 6.3 and pK_i_ = 6.2, respectively) [50].

One of the typical drugs that has been used for almost 50 years for the treatment of pituitary tumors, Parkinson’s disease, hyperprolactinemia, neuroleptic malignant syndrome, and, as an adjunct, type 2 diabetes is an ergoline derivative and dopamine agonist bromocriptine. Typically, this drug has many targets of actions: 5-HT_1D_ receptor (acts as partial agonist) with pK_i_ = 8.0 [50], α_2A_-AR (acts as antagonist) with pK_i_ = 8.0 [50], 5-HT_1A_ receptor (acts as partial agonist) with pK_i_ = 7.9 [50], D_2_ DR (acts as full agonist [50]; however, in rats it is a partial agonist [7]) with pK_i_ = 7.3–8.3, 5-HT_7_ receptor (acts as full agonist) with pK_i_ = 7.3–8.0 [68], D_3_ DR (acts as partial agonist [50]; however, in rats it is a full agonist [7]) with pK_i_ = 7.1–8.2 [50], α_2C_-AR (acts as antagonist) with pK_i_ = 7.6, 5-HT_6_ receptor (act as full agonist [69]; however, in rats it is a partial agonist [70]) with pK_i_ = 7.5, α_2B_-AR (acts as antagonist) with pK_i_ = 7.5 [50], 5-HT_2B_ receptor (act as antagonist) with pK_i_ = 7.3 [50], 5-HT_2A_ receptor (act as partial agonist [50]) with pK_i_ = 7.0, Other receptors (5-HT_1B_ receptor, D_4_ DR, D_5_ DR, D_1_ DR, and 5-HT_2C_ receptor reveal lower affinity with pK_i_s < 7.0 [50]). When applied to experimental animals one should count all effects listed above.

The drug with declared multiple effects is apomorphine, historically used to relieve anxiety and craving in alcoholics, as an emetic, or in treating erectile dysfunction. Currently, apomorphine is used in the treatment of Parkinson’s disease but should be used together with antiemetics. Contrary to its name, apomorphine does not contain morphine or its skeleton, nor does it bind to opioid receptors. It is declared as a non-selective dopamine agonist which activates both D_2_-like and, to a much lesser extent, D_1_-like receptors, an antagonist of 5-HT_2_ and α-AR with high affinity. In detail, D_4_ DR binds this compound as a partial agonist with pK_i_ = 8.4 [50], rat and human D_3_ DR binds this compound as a partial agonist with pK_i_ = 7.7 [7], and with pK_i_ = 6.1–7.6 [50], respectively. Rat and human D_2_ DRs bind this compound as a partial agonist with pK_i_ = 7.6 [7], and pK_i_ = 5.7–7.5 [50], respectively. α_2C_-AR binds this compound as an antagonist with pK_i_ = 7.4 [50], α_2B_-AR binds this compound as an antagonist with pK_i_ = 7.2 [50], D_5_ DR binds this compound as a partial agonist with pK_i_ = 6.4–7.8 [50], 5-HT_2C_ receptors bind this compound as an antagonist with pK_i_ = 7.0 [50], 5-HT_1A_ receptors bind this compound as a partial agonist with pK_i_ = 6.9 [50], 5-HT_2A_ receptor binds this compound as an antagonist with pK_i_ = 6.9 [50], 5-HT_2B_ receptor binds this compound as an antagonist with pK_i_ = 6.9 [50], α_2A_-AR binds this compound as a partial agonist with pK_i_ = 6.9 [50]. All these values, except stated otherwise, come from human receptors.

Benzquinamide is more potent inhibitor of cyclooxygenase COX-2 (pIC_50_ = 8.3) than agonist on D_2_ DR (pK_i_ = 5.4) [71]. 

## 3. DR Antagonists

### 3.1. So-Called Selective Dopamine Receptor Antagonists

An example of a drug declared as D_1_ (or D_1_-like family, pK_i_ = 8.4 for D_1_ DR) selective antagonist is flupentixol [13]. However, this antagonist also affects σ_3_-receptors [72] (pK_i_ = 8.86). In addition to that, this ligand also antagonizes the D_2_-like family (pK_i_ = 8.82 for D_2_ DR, and pK_i_ = 8.96 for D_3_ DR, respectively) [73].

Another example of a drug, declared as specific, is L-741,626 which is usually marked as a potent D_2_ DR selective antagonist over D_3_ DR and D_4_ DR, respectively (D_2_ DR: pK_i_ = 7.95–8.35 [74], D_3_ DR: pK_i_ = 6.79–7.04 [74], D_3_ DR: pK_i_ = 5.82 [74]). However, this compound also binds to the σ-1 receptor with pK_i_ = 7.71 [75]. 

Domperidone, acting peripherally, as it is extensively metabolized in the liver, and has the low central nervous system penetration, is the next example of a declared specific D_2_ and D_3_ DR antagonist (pK_i_ = 7.9–8.4, and pK_i_ = 7.1–7.6, for D_2_ and D_3_ DRs, respectively [73]) is also able to bind to 5-HT_3A_/5-HT_3B_ receptors with pK_IC50_ = 7.0 [76]. 

Nafadotride is usually considered a highly potent and competitive, centrally active D_3_ DR antagonist (pK_i_ = 9.5 [77]) over D_2_ DR (pK_i_ = 8.8 [77]) and mainly over D_4_ DR (pK_i_ = 6.4 [64]). However, also 5-HT_1A_ receptor can be activated (full agonisms exist here [78]) by this drug with pK_i_ = 7.3.

PG01037 is considered as D_3_ DR selective antagonist (pK_i_ = 9.2 [79]). Some other papers indicate different affinity (from pK_i_ = 8.68 [80]to pK_i_ = 9.5 [81]), and some indicate significant affinity to D_2_ DR (pK_i_ = 7.13 [81]), to 5-HT_2C_ (pK_i_ = 7.33 [79]), to 5-HT_2A_ (pK_i_ = 7.2 [79]), and to 5-HT_1A_ (pK_i_ = 7.07 [79]). 

The specific situation comes with spiperone. Spiperone is considered a D_2_-like dopamine receptor-specific ligand (pK_i_ = 8.4–9.4 [82], 9.2 [83], and 9.3 [82] for D_2_, D_3_, and D_4_ DR, respectively) and is commercially available as a tritiated ligand. However, this ligand also exhibits similar affinities (pK_i_ = 7.8–9.4) for 5-HT_2A_ receptors [84], 5-HT_1B_ receptors (pK_i_ = 8.3) [85], and α_1A_, α_1B_ and α_1D-_AR_s_ (pK_i_ = 8.3, 9.2, and 8.1, respectively) [86]. This is a very inconvenient feature as tritiated spiperone (^3^H-spiperone) is very often used as a specific ligand for binding of D_2_-like family: we found 1,156 results for ^3^H-spiperone in a Pubmed search (accessed on 21 March 2022). One should be cautious when interpreting the results obtained with ^3^H-spiperone in the cerebral cortex, striatum, olfactory tubercle, substantia nigra, globus pallidus, nucleus accumbens, CA1 region of hippocampus, hypothalamus, and cerebellum (see Table 2 for the presence of specific 5-HT subtypes). Moreover, the pK_i_s of D_1_ and D_5_ DRs are 6.7, and 5.4, respectively [23]. 

On the other hand, another radiolabeled ligand, raclopride is specific for DR and has a similar affinity to D_2_ DR (pK_i_ = 7.77 [87]) and D_3_ DR (pK_i_ = 7.82 [87]) but do not bind significantly to D_4_ DR (pK_i_ = 5.51 [87]) and also not to D_1_ DR (pK_i_ = 4.43 [87]).

Another radiolabeled ligand used in DR assays, 7-OH-DPAT, binds to D_3_ DR with pK_i_ = 5.85–9.6 [88,89]. It is necessary to say that the study with pK_i_ = 5.85 [88] is exceptional, and usually, the pK_i_ rank is between 8 and 9. The affinity to D_2_ DR is lower (pK_i_ = 6.51 [90]-8.73 [91], as well as to D_4_ DR (pK_i_ = 6.83 [92]). Besides these receptors, 7-OH-DPAT has also some affinity to 5-HT_1A_ receptors (pK_i_ = 7.33 [92]), and σ1-receptors (pK_i_ = 7.63 [93]).

### 3.2. Drugs–Dopamine Receptor Antagonists with Multiple Targets of Action 

Similar to agonists, there are some drugs used in the treatment of psychiatric/neurological disorders with multiple targets action. One of them is blonanserin, an atypical antipsychotic for the treatment of schizophrenia [94]. The spectrum of targets is relatively close, but in addition to D_2_ DRs (pK_i_ = 9.9 [95]) it also antagonize the action on 5-HT_2A_ receptors (pK_i_ = 9.1 [95]) and on D_3_ DRs (pK_i_ = 6.3 [96]). Blonanserin has a low affinity [97] for 5-HT_2C_, α_1_-ARs, histamine H_1_, and M_1_ muscarinic receptors but displays a relatively high affinity for 5-HT_6_ receptors (pK_i_ = 7.93) [97].

Another atypical antipsychotic drug, risperidone, binds to 5-HT_7_ receptor in rat as an inverse agonist with pKd = 8.9–9.0 [98], to 5-HT_2A_ receptor as an inverse agonist with pK_i_ = 9.3–10.0 [67], to D_2_ DR as an antagonist with pK_i_ = 9.4 [99], to 5-HT_2A_ receptor in rat as an antagonist with pK_i_ = 8.5 [100], to 5-HT_7_ receptor as an inverse agonist with pK_i_ = 8.3–8.7 [101], to α_1A_-AR as an antagonist with pK_i_ = 8.4 [86], to α_1B_-AR as an antagonist with pK_i_ = 8.0 [86], to α_2C_-AR as an antagonist with pK_i_ = 8.49 [102], to α_2A_-AR as an antagonist with pK_i_ = 8.0 [102], to 5-HT_1D_ receptor as an antagonist with pK_i_ = 7.8–8.0 [103], to H_1_ histamine receptor as an antagonist with pK_i_ = 7.6–7.8 [67,103], to 5-HT_2C_ receptor as an inverse agonist with pK_i_ = 7.5–7.6 [67], to 5-HT_2B_ receptor as an antagonist with pK_i_ = 7.7 [104], to 5-HT_1A_ receptor as an antagonist with pK_i_ = 7.68 [105], to α_1D_-adrenoceptor as an antagonist with pK_i_ = 7.4 [86], to D_3_ DR as an antagonist with pK_i_ = 7.0 [106], and to 5-HT_1B_ receptor as antagonist with pK_i_ = 6.6–7.3 [103]. Other targets (5-HT_6_, 5-HT_1F_ receptors) have a lower affinity (pK_i_ less 7.0).

Perphenazine, a typical antipsychotic, binds to a set of receptors: to D_2_ DR as an antagonist with pK_i_ = 8.9–9.6 [67], to 5-HT_2A_ receptor as an antagonist with pK_i_ = 8.2 [67], to H_1_ histamine receptor as an antagonist with pK_i_ =8.1 [67], to other 5-HT receptors (5-HT_6_, 5-HT_7_, 5-HT_2C_) the pK_i_ vary between 7.8 and 6.9 [67,98,107].

Trifluoperazine, a typical antipsychotic drug, binds to D_2_ DR as an antagonist with pK_i_ = 8.9–9.0 [67], to 5-HT_2A_ receptor as an antagonist with pK_i_ = 7.9 [67], to D_4_ DR as an antagonist with pK_i_ = 7.4 [108], and to H_1_ histamine receptor as an antagonist with pK_i_ = 7.2 [67].

Quetiapine, an anti-psychotic drug, is bound with the highest affinity by the H_1_ histamine receptor as an antagonist with pK_i_ = 8.0–8.7 [67]. Lower affinity (antagonistic) is revealed by D_2_ DR (pK_i_ = 7.2) [99]. Similar affinity as in D_2_ DR have 5-HT_2A_ (pK_i_ = 6.4–7.0, [67,103]) and 5-HT_1A_ (pK_i_ = 6.5–7.1, [103,104]) receptors. Interestingly, this drug can behave as an agonist [103] or as an antagonist [67] on 5-HT_2A_ receptors. In addition to that, it also binds to α_2C_-AR as an antagonist with pK_i_ = 7.0 [109], to α_1A_-AR, and α_1B_-AR as an antagonist with pK_i_ = 7.0 [109], and M_1_ muscarinic receptors as an antagonist with pK_i_ = 7.0–7.25 [105,110].

The typical antipsychotic drug, haloperidol, has a wide spectrum of actions, including antagonism on DRs (D_4_ DR pK_i_ = 8.7–8.8, D_2_ DR pK_i_ = 7.4–8.8, D_3_ DR pK_i_ = 7.5–8.6, D_1_ DR pK_i_ = 7.6–8.2) and antagonism on 5-HT receptors (5-HT_2A_ receptor pK_i_ = 6.7–7.3, other 5-HT receptors (5-HT_1D_, 5-HT_7_) have pK_i_ < 7.0). Similarly, D_5_ DR and H_1_ histamine receptors reveal pK_i_ < 7.0. Relatively high affinity to this drug also reveal α_1A_-AR (antagonist, pK_i_ = 7.89–8.55 [111,112]), α_1B_-AR (antagonist, pK_i_ = 8.00 [86]), α_1D_-AR (antagonist, pK_i_ = 7.4 [86]), α_2A_-AR (antagonist, pK_i_ = 7.6 [111]), and α_2C_-AR (antagonist, pK_i_ = 7.6 [109]).

Sertindole is an atypical antipsychotic drug with high affinity to 5-HT_2A_ receptor (antagonist, pK_i_ = 9.2–9.4 [67]), to 5-HT_2C_ receptor (inverse agonist, pK_i_ = 9.0–9.2 [67]), to D_2_ DR (antagonist, pK_i_ = 8.0–8.9 [67]), to α_1A_-AR (antagonist, pK_i_ = 9.43 [113]), to α_1B_-AR (antagonist, pK_i_ = 9.48 [113]), to α_1D_-AR (antagonist, pK_i_ = 9.18 [113]), to H_1_ histamine receptor (antagonist, pK_i_ = 9.29 [114]), and to D_4_ DR (antagonist, pK_i_ = 7.8–9.1 [108]). Relatively high affinity reveal D_3_ DR (antagonist, pK_i_ = 8.0–8.8 [103]), Kv11.1/HERG kalium channels (antagonist, pK_IC50_ = 8.57 [115]), 5-HT_6_ (antagonist, pK_i_ = 8.3 [116]), and D_1_ DR (antagonist, pK_i_ = 7.92 [117]). Possible targets are 5-HT_1D_ receptor (antagonist, pK_i_ = 7.2 [103]) and 5-HT_1B_ receptor (antagonist, pK_i_ = 7.0 [103]).

Loxapine is a typical antipsychotic drug that binds to a wide spectrum of targets: H_1_ histamine receptor, where it acts as an antagonist with pK_i_ = 8.2 [67], D_2_ DR, where it acts as an antagonist with pK_i_ = 7.9–8.3 [67], D_4_ DR, where it acts as an antagonist with pK_i_ = 8.1, 5-HT_2A_ receptor [108], where it acts as an inverse agonist with pK_i_ = 8.1 [67], 5-HT_2C_ receptor, where it acts as an inverse agonist with pK_i_ = 7.8–8.0 [67], D_3_ DR, where it acts as an antagonist with pK_i_ = 7.7 [118], 5-HT_6_ receptor, where it acts as an inverse agonist with pK_i_ = 7.4–7.6 [107], 5-HT_7_ receptor, where it acts as an antagonist with pK_i_ = 6.8–7.4 [98]. 

Domperidone is declared as an orally active, peripherally acting, and selective antagonist of dopamine D_2_ and D_3_ DR. Although the selectivity to these receptors is quite well (pK_i_ = 7.9–8.4, pK_i_ = 7.1–7.6, respectively [73]), domperidone can also antagonize 5-HT_3_ receptors with pK_IC50_ = 7.0 [76].

Promazine, a phenothiazine antipsychotic, binds not only to D_2_ DR and D_3_ DR (pK_i_ = 6.5 and 6.8, respectively [119]) but also with similar, although not very high, affinity to H_1_ histamine receptors (pK_i_ = 5.9 [120]). 

## 4. Discussion

**Figure 1:**
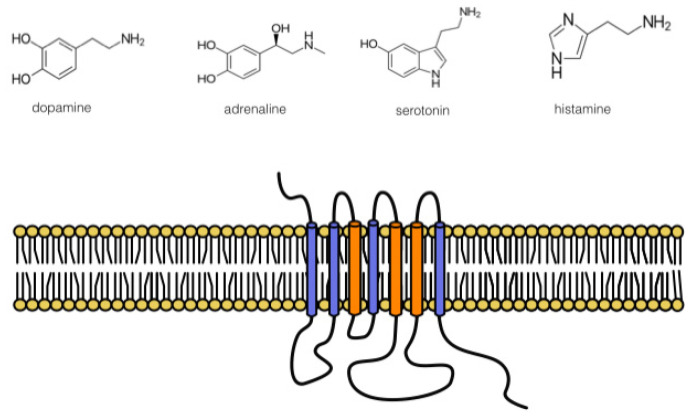
Schematic structure of DR and above, potential ligands. Transmembrane zones important to dopamine binding are shown in orange (see text for details).

The first thing that should be discussed is the similarity in the amino acid binding pocket of DRs with α_2_-ARs and 5-HT receptors. It is possible to deduce this statement from apparently similar affinities (pK_i_s) for dopamine as given in the Introduction. This is given by the similarity of neurotransmitter structures: noradrenaline, adrenaline, dopamine, and serotonin (see Figure 1). However, as mentioned above, the main role plays in the relationship between specific G protein-coupled receptors, i.e., the sequence homology in the binding pocket between dopamine, serotonin receptors, and adrenoceptors. These homologies have been well documented for the second extracellular loop, as discussed in [121].

A second fact that implies the similarities in binding pocket/amino acid homology is that other ligands that bind to the similar amino acid residues in DRs as dopamine would also affect 5-HT receptors and α_2_-ARs. The examples of such ligands were listed above both for agonists and antagonists.

In general, the length, organization, and amino acid homology in the D_1_-like DR subfamily is quite high [122]. This is the reason for so far not synthesizing specific agonists to D_5_ DR (see below). The D_1_-like DRs have a shorter third intracellular loop and a longer carboxy-terminus compared to the D_2_-like DR subtypes [122]. The third intracellular loop and carboxy-terminus are not structures responsible for binding. The third intracellular loop and a carboxy-terminus play a role in the G protein binding. The receptor regions responsible for binding are transmembrane zones. More precisely, the predicted binding site of dopamine in D_2_ DR is located in the top third of the 7-TM barrel involving TM domains 3–6 [123]. These authors also divided dopamine ligands into two groups according to their binding properties: first, clozapine-like bulky antagonists; and second, ligands with two aromatic or ring moieties connected by a flexible linker with a protonated amine group as in haloperidol [123]. The first group occupies the region between TM3, TM4, TM5, and TM6 (the agonist binding pocket), and the second group occupies the region between TM2, TM3, TM6, and TM7, with minimal contact with TM4 and TM5 [123]. The binding pocket of D_1_ DR is slightly different comprising TM6, extracellular loop 2, TM5, and TM3 [121].

D_3_ DR and D_2_ DR subtypes have substantial amino acid sequence homology [122]. 

The main aim of this review is to show that drugs declared by manufacturers as specific could be, in some cases, able to bind to other targets than to DRs. This can produce ambiguous results. Importantly, there are enough ligands with sufficient specificity for DR subtypes (see Table 1). The interested researcher should search available databases carefully for the ligands with well-documented selectivity to specific DR subtypes and no to rely on the information from the manufacturer. 

Nevertheless, one can experience different values for the same compound. As reported here, the affinities of 5-HT_1A_, 5-HT_2C_ serotonin receptors, and β_1_-ARs to A68930 are similar to those of D_1_-like DRs (when considering the data from [47]), but the data from [46] are quite similar. Another example reported here is SKF 38393. The pK_i_ values differ according to specific references in humans [13,23,30], which also vary from this value in rats. This can originate from different experimental conditions (temperature, incubation time, tissue, cell culture properties, and others). In such a case, one should be cautious with the selection of this compound for subtype determination or interpretation of results obtained with this drug in the literature. If possible, it is recommended to avoid such ligands.

However, the nature of drug properties reviewed here could be more complex. One should also consider the anatomical relationship between the terminals that release dopamine and other receptors—this concerns both 5-HT receptors and α_2_-ARs. Dopamine terminals are frequently localized in tight contact with other axons configuring a triad—a configuration in which a neuron is connected to both the pre-synaptic element and post-synaptic (usually dendritic) target. Triads are common in the hippocampus, striatum, and medial frontal cortex (for a review, see [124]). These triads can contain both dopamine and serotonin or adrenergic terminals. The first point on how the interaction between DRs and 5-HT receptors can occur is the formation of the heteroreceptor complexes of D_2_ DR and 5-HT_2A_ receptors [125]. The heterocomplexes could explain the effects of atypical antipsychotic drugs [125]. One of the possible mechanisms is based on blocking the allosteric enhancement of D_2_ DR protomer signaling by 5-HT_2A_ receptor protomer activation. Another mechanism by which dopamine can interact with serotonin is the release of L-DOPA as a “false (or substitute)” neurotransmitter in the serotonin synapse [126]. “False neurotransmitter” is considered as an ectopic neurotransmitter in a neuron, which replaces the normal neurotransmitter in storage vesicles. When it is the case of L-DOPA it is then able to increase the dopamine levels as L-DOPA is a dopamine precursor. Moreover, dopamine can also act as a “false neurotransmitter” in noradrenergic neurons [126].

Another aspect is given by the presence (although sometimes doubted in dopaminergic synapse) of volume transmission [127,128,129]. This type of connection allows the spreading of the neurotransmitter to a higher distance (more than 10 μm in comparison to 30–40 nm in classical synapse), affecting 200 other dopamine synapses instead of only one post-synaptic membrane in the classical synapse. This can further be the factor of cross action of dopamine.

On the other hand, we cannot consider this a problem; this is most probably the physiological role of the transmitter.

It can be deduced from Table 1 that a D_5_ DR agonist does not exist to date and that the selectivity of the antagonist comprises the other member of D_1_-like family—D_1_ DR. However, specific agonists (A77636, SKF-81297, and SKF-83959) exist for D_1_ DR. Thus, it is possible to distinguish between D_1_ DR and D_5_ DR using the D_1_ DR agonists. 

Specific subtypes in the D_2_-like family can be distinguished using specific antagonists for D_2_ DR (pipotiazine, ML321), D_3_ DR (S33084, SB 277011-A, (+)-S-14297), and D_4_ DR (sonepiprazole, L745870, A-381393, L741742, ML398). One should also consider the presence of off-targets (Table 2) when evaluating the role of specific dopamine receptors, as some receptors have a lower affinity to relatively selective ligand, but if the density of off-target receptors is much higher than DR that the proportion of the binding could be shifted.

Even though the attribution of a drug to be DR agonist/antagonist can also be the result of the side effect on another receptor. Thus, some drugs can primarily bind to other receptors and also reveal dopaminergic action. Examples of such drugs are some antipsychotics listed above (bromocriptine acting mainly at 5-HT receptors [50], risperidone acting mainly at 5-HT receptors [67,98], quetiapine which is H_1_ histamine receptor antagonist [67], sertindole which has a high affinity to 5-HT receptors [67], and loxapine acting on H_1_ histamine receptors [67]). Other drugs that could bind to DRs as to “second target” are muscarinic receptor agonists AC-260584, 77-LH-28-1, and LY-593039, which bind similarly to M_1_ muscarinic receptors and to D_2_ DRs [130]. Another group of drugs binds primarily to 5-HT receptors. An example of such a drug is 8-OH-DPAT (the binding of related 7-OH-DPAT is mentioned above), which is used in the tritiated form as a radioligand for 5-HT receptors. [^3^H]8-OH-DPAT binds to 5-HT_1A_ receptors with high affinity (pK_i_ = 9.33 [131]). The affinity of 5-HT_1B_ receptors is lower (pK_i_ = 6.25 [132]) and corresponds to the affinity to DR (pK_i_ = 7.07 [133]). Another compound acting on 5-HT receptors and with similar binding to DRs is iloperidone, an atypical antipsychotic drug. This compound binds to 5-HT_1A_, 5-HT_6_, and 5-HT_7_ receptors with pK_i_ = 6.8–7.7 [134,135] and to D_2_ DR with pK_i_ = 7.0 [136]. Another atypical antipsychotic drug zotepine has antagonistic activity at 5-HT receptors (5-HT_1D_ pK_i_ = 9.3 [103], 5-HT_2A_ pK_i_ = 8.6 [103]) and on D_2_ DR (pK_i_ = 8.0 [103]), D3 DR (pK_i_ = 8.2 [103]), D4 DR (pK_i_ = 7.4 [103]). Besides that, zotepine also binds to H_1_ histamine receptors (pK_i_ = 9.2 [103]) and to 5-HT_6_ and to 5-HT_7_ with pK_i_ = 8.9, and pK_i_ = 8.8, respectively [98]. These examples just illustrate the complexity of the cross bunding between drugs suggested to be selective to specific receptors. The number of such interactions would increase with the increase in our knowledge on this topic.

This review can also help with the interpretation of results obtained with antipsychotic drugs as it critically reviews the real binding to different targets, and the reader can compare the affinities of specific target molecules to these ligands. In Table 2 it is possible to find the presence of other receptors (subtypes of α_2_-ARs and 5-HT receptors) that can help the interpretation of data obtained with antipsychotic drugs.

We can conclude that one should be very cautious when selecting the DR ligand with the aim to determine the role of a specific DR subtype in studied CNS function. This review can help in such selection. Not only the selectivity but also the presence of typical off-targets to dopamine ligands (subtypes of α_2_-ARs and 5-HT receptors) should be considered, and finally, the new research can bring new knowledge, and the supposed selectivity of the specific ligand could be doubted.

## Figures and Tables

**Table 1:** Selective ligands to dopamine receptor subtypes. Listed are both subtype(s) and family-specific compounds.

	D_1_	D_2_	D_3_	D_4_	D_5_
**agonist**	A77636 SKF-81297 SKF-83959	MLS1547 ^2^ Rotigotine ^3^ Ropinirole ^4^ Pramipexole ^4^ PD 128907 ^4^ PD168,077 ^7^ A412997 ^8^	Rotigotine ^3^ Ropinirole ^4^ Pramipexole ^4^ PD 128907 ^4^ A412997 ^8^ [^3^H]PD128907 ^9^	Rotigotine ^3^ PD168,077 ^7^ A412997 ^8^	
**antagonist**	SKF-83566 ^1^ SCH-23390 ^1^ Ecopipam ^1^ [^125^I]SCH23982 ^1,9^	pipotiazine perospirone ^5^ raclopride ^3^ ML321 Prochlorperazine ^4^ Sulpiride ^5^ NGB 2904 ^6^	Perospirone ^5^ Raclopride ^3^ Prochlorperazine ^4^ Sulpiride ^5^ S33084 NGB 2904 ^6^ SB 277011-A (+)-S-14297	Perospirone ^5^ Sulpiride ^5^ sonepiprazole L745870 A-381393 L741742 ML398 [^125^I]L750667 ^9^ [^3^H]NGD941 ^9^	SKF-83566 ^1^ SCH-23390 ^1^ Ecopipam ^1^ [^125^I]SCH23982 ^1,9^

^1^ The selectivity is expressed to D_1_-like DRs. ^2^ Biased D_2_ DR agonist [38]: it antagonizes arrestin recruitment to D_2_ DR but behaves as an agonist in its capacity to induce D_2_ DR signaling. ^3^ D_2_ DR and D_3_ DR selective over D_4_ DR. ^4^ D_2_ DR and D_3_ DR selective. ^5^ The selectivity is expressed to D_2_-like DR. ^6^ D_3_ DR selective over D_2_ DR. ^7^ Slightly more selective to D_4_ DR than to D_2_ DR. ^8^ Selectivity D_4_ DR > D_3_ DR > D_2_ DR. ^9^ Please note that this is radioligand.

**Table 2:** The co-presence of receptor types in specific brain areas.

CNS Area		DR Presence	α_2_-AR Presence	5-HT Presence
Cerebral cortex		D_1_-like D_2_-like	α_2C_-AR	5-HT_2_ 5-HT_4_ 5-HT_6_ 5-HT_1A_ 5-HT_1B_ ^5^ 5-HT_1E_ 5-HT_1F_ 5-HT_5A_
Amygdala		D_1_-like	α_2C_-AR α_2A_-AR^2^	5-HT2C 5-HT_6_ 5-HT_1B_ ^5^
Substantia nigra	pars compacta	D_2_ DR	α_2C_-AR	5-HT_4_ 5-HT_1B_ ^6^ 5-HT_1D_ ^6^ 5-HT_1F_ ^6^
	pars reticularis			5-HT_4_ 5-HT_1B_ ^6^ 5-HT_1D_ ^6^ 5-HT_1F_ ^6^
Striatum (Caudate-putamen)		D_1_ DR D_2_ DR D_3_ DR	α_2C_-AR ^1^	5-HT2A/2C 5-HT_4_ ^1^ 5-HT_6_ 5-HT_1B_ ^2^ 5-HT_1D_ ^1^ 5-HT_1F_ ^7^
Globus pallidus		D_2_-like	α_2C_-AR ^1^	5-HT_4_ ^1^ 5-HT_1B_ 5-HT_1D_ ^1^ 5-HT_1F_
Ncl. accumbens		D_1_ DR		5-HT2A/2C 5-HT_6_ 5-HT_1B_ ^2^
Hippocampus (without further specification)		D_5_ DR D_4_ DR	α_2C_-AR α_2A_-AR ^2^ α_2B_-AR ^2^	5-HT_4_ 5-HT_6_ 5-HT_7_ 5-HT_1A_ 5-HT_1F_ 5-HT_5A_ ^2^
CA1		D_1_-like D_2_-like		5-HT_4_ 5-HT_1A_ 5-HT_1B_ ^2^ 5-HT_1E_ 5-HT_5A_ ^2^
CA3		D_1_-like D_2_-like		5-HT_1E_ 5-HT_5A_ ^2^
Thalamus		D_1_ DR	α_2B_-AR α_2C_-AR^2^	5-HT_2A_ 5-HT_6_ 5-HT_7_
Ncl. subthalamicus		D_1_ DR	α_2C_-AR ^1^	5-HT_1B_ ^2^
Hypothalamus		D_5_ DR D_3_ DR	α_2A_-AR ^2^	5-HT2C ^3^ 5-HT_6_ 5-HT_7_ ^4^ 5-HT_1A_ 5-HT_1B_ ^5^ 5-HT_5_ ^4^
Olfactory tubercle		D_3_ DR	α_2C_-AR	5-HT2A/2C 5-HT_6_
Midbrain		D_4_ DR	α_2A_-AR ^2^ α_2C_-AR ^2^	
Ventral tegmental area		D_2_ DR		
Cerebellum		D_3_ DR D_4_ DR	α_2A_-AR ^2^ α_2B_-AR ^2^	5-HT_6_ 5-HT_1B_ ^2^ 5-HT_5A_ ^2^

The presence of specific receptor types was referred to by [1,40,41,42,43,44]. D_1_-like means the presence of D_1_ DRs and D_5_ DRs,.D_2_-like means the presence of D_2_ DRs, D_3_ DRs, and D_4_ DRs. The presence of receptors in the cerebral cortex can be more specific to layers, part of the cortex, etc. Please see [1,40,41,42,43,44] for detail. ^1^ Referenced as a presence of subtype in basal ganglia (no further specification). ^2^ mRNA expression only does not necessarily mean the presence of receptors binding sites. ^3^ Specifically in the dorsomedial hypothalamus and the paraventricular nucleus. ^4^ Generally in the hypothalamus, specifically in the suprachiasmatic nucleus. ^5^ Low autoradiography detected levels. ^6^ Referenced as a presence of subtype in substantia nigra (no further specification). ^7^ Specifically in the putamen.

## Data Availability

Not applicable.

## Abbreviations

List of abbreviations and a short explanation of the terms used.

**Abbreviation**	**Explanation**
DR(s)	Dopamine receptor(s)
ARs	Adrenoceptors
5-HT	Serotonin
TM	Transmembrane zone
pK_i_	The negative logarithm of the K_i_ value (the molar concentration of the competing ligand that would occupy 50% of the receptors)
pK_D_	The negative logarithm of K_D_ value (the equilibrium dissociation constant represents the concentration of radioligand occupying half of the maximum receptor population)
pA_2_	The measure of the potency of an antagonist, negative logarithm of the molar concentration of an antagonist that would produce a two-fold shift in the concentration-response curve for an agonist
pEC_50_	The negative logarithm of EC_50_ value (the molar concentration of an agonist that produces 50% of the maximum possible response for that agonist). This value can vary when comparing different activation pathways
